# Discriminant compounds and a predictive S-score for the manual grading standard of *Jasminum sambac*

**DOI:** 10.1038/s41598-026-57149-2

**Published:** 2026-06-22

**Authors:** Jiping Wang, Ye Zhang, Tiedong Lu, Zhongyi Li, Tianming Su, Tieguang He

**Affiliations:** Institute of Agricultural Resources and Environment Research, Guangxi Key Laboratory of Arable Land Conservation, Guangxi Academy of Agricultural Sciences, Nanning, 53007 Guangxi China

**Keywords:** Commercial jasmine grade, Volatile organic compounds, Quality grading, S-score, Ecology, Ecology, Plant sciences

## Abstract

Jasmine (*Jasminum sambac*) is a highly valued aromatic plant, widely used for scenting tea and extracting essential oils. Flower quality directly influences the market value of these products. However, the classification of jasmine flowers currently relies on empirical visual inspection and mechanical sieving, lacking objective chemical markers. In this study, freshly picked commercial jasmine buds (grade A and grade B) and fully bloomed flowers (grade I and grade II) were analyzed to examine differences in their volatile compound profiles using headspace solid-phase microextraction (HS-SPME) coupled with gas chromatography-mass spectrometry (GC-MS). According to the general commercial grading standards used by growers and tea companies, 25 compounds differed significantly between grade A and grade B buds, while 134 compounds differed significantly between grade I and grade II opened flowers (*P* < 0.05). Intersection analysis identified six compounds common across grade comparisons. By comprehensively evaluating their fold changes and relative abundances, three compounds—(*E*)-3-Hexen-1-ol, Caryophyllene, and (*E*,*E*)-3,5-Octadien-2-one—were selected as potential markers. Based on the current dataset, the S-score provides an objective, data-driven approach to distinguish jasmine flower grades. However, as a preliminary model, its proposed threshold (S > 9.5) requires independent validation using samples from different seasons, regions, or production batches before any practical application.

**Supplementary Information:**

The online version contains supplementary material available at https://doi.org/10.1038/s41598-026-57149-2.

## Introduction

*Jasminum sambac* (L.) Aiton is a shrub species belonging to the Oleaceae family^1^. Valued for its unique and captivating fragrance as well as high ornamental value, jasmine is extensively cultivated in regions such as Hengxian County, Qianwei, and Fuzhou in China^2^. Owing to its distinctive geographical and natural conditions, Guangxi Hengxian has become the world’s largest jasmine planting area^3^. Hengxian jasmine is renowned for its long flowering period, large bud size, high yield, excellent quality, and intense aroma, accounting for over 80% of China’s total jasmine production. It is recognized as the largest jasmine production and processing base globally and has been designated the “Hometown of Jasmine in China” by the State Forestry Administration and the China Flower Association. Jasmine flowers are primarily used for scenting tea and extracting essential oils, with the highest-quality flowers typically reserved for jasmine tea production^4–6^.

Jasmine is categorized into single-petal and double-petal varieties^2,7^. Due to its strong fragrance and desirable flower form, double-petal jasmine is widely cultivated in Hengxian^8^. Buds are picked during the daytime under fair weather conditions, spread on clean ground at a thickness of approximately 15–20 cm, and turned periodically to promote uniform blooming. The buds generally open between 20:00 and 22:00 on the same day. Fully opened flowers are then separated using a specialized sieving device and classified as grade I^9^. Unopened or partially opened blooms are graded as grade II, while the remainder are discarded.

Volatile components in jasmine are key indicators of its commercial value^10,11^. Previous studies have shown that the release of aroma compounds peaks after flowering^12^. The composition and concentration of these aroma components directly influence the quality of jasmine tea^11,13^. Thus, the availability of high-quality jasmine flowers rich in aromatic compounds is essential. In large-scale production of jasmine floral condensates, Linalool, Indole, and Methyl anthranilate have been identified as characteristic odorants^14,15^. For jasmine tea evaluation, an index known as the “jasmine tea flavor” (JIF) incorporates *α*-Farnesene, (*Z*)−3-Hexenyl benzoate, Methyl anthranilate, Indole, and Linalool^16^.

Although previous research has focused on extracting aroma components and grading jasmine tea^6,11^, the classification of jasmine flowers—particularly buds and opened flowers—remains unclear. In practice, growers and tea companies employ a set of empirical standards to evaluate flower quality based on plant growth and bud maturity, which in turn determines the price. Additionally, mechanical sieves are used to grade opened flowers. However, these methods rely primarily on visual inspection and sensory evaluation, lacking a rational, data-driven assessment system. The objective of this study was therefore to identify volatile organic compounds (VOCs) that differ between commercial grades of jasmine buds and opened flowers, and to evaluate whether specific compounds could serve as objective discriminant markers for quality grading.

Investigating and analyzing the differences in aroma components among commercially graded jasmine flowers under existing classification schemes, and identifying signature differential compounds as grading markers, are important steps toward standardizing jasmine flower classification and promoting selective breeding and industrial development. Gas chromatography-mass spectrometry (GC-MS) is a well-established technique for VOC detection and has been widely applied to analyze aroma components in flowers, wines, and other materials^17–19^. In this study, headspace microextraction was used to extract aroma compounds from jasmine samples, followed by GC–MS analysis to identify differential components among different flower grades. This approach aimed to reveal correlations between grades and changes in aroma profiles after harvesting. By identifying key aroma components that distinguish flower grades before scenting and that differentiate flowers from superior versus ordinary growth conditions during picking, this study provides a reference for improving jasmine grading and optimizing cultivation practices.

## Materials and methods

### Sample collection

Fresh flower samples of double-petal jasmine were collected from Hengxian, Guangxi Province, China (108° 48′–109° 37′ E, 22° 08′–22° 30′ N) on 23 July 2020. Prior to sampling, planting areas and plant growth were surveyed. A total of 11 sampling sites were selected, representing plots with good (grade A) and normal (grade B) plant growth. Flowers from corresponding plots were collected at each site. Each sample consisted of 10–15 commercial jasmine buds, which were stored and transported in liquid nitrogen for subsequent GC–MS analysis. In total, 22 independent biological samples (11 sites × 2 grades) were analyzed without pooling.

Another set of jasmine flowers was collected from Hengxian HuaCheng Tea Co., Ltd. Fresh jasmine buds were spread on the ground at the tea factory until they opened. The opened flowers were then sieved using a double-layer sieve with pore sizes of 10 × 10 mm and 5 × 20 mm. Flowers retained by the first sieve were collected as grade I; those passing through the first sieve but retained by the second were sampled as grade II. Grade I flowers are used for tea scenting, while grade II flowers are generally used for other purposes such as essential oil extraction. three samples were taken from each grade, with each replicate consisting of 10–20 flowers pooled. The plant materials were identified as *Jasminum sambac* (L.) Aiton by Prof. Riyuan Chen, South China Agricultural University. A voucher specimen (specimen no. Jsd20200723-001) was deposited at the lab in Institute of Agricultural Resources and Environment Research, Guangxi Academy of Agricultural Sciences. All plant materials were collected under permission from the landowners and the tea company, and no endangered or protected plant species were involved in this study.

### Metabolite extraction and GC–MS analysis

A 300-mg flower sample was placed in a 20-mL headspace vial, and 10 µL of 2-octanol (10 mg L⁻¹ stock in deionized water) was added as an internal standard. All samples were analyzed by gas chromatography–mass spectrometry (GC–MS).

Headspace solid-phase microextraction (HS-SPME) was performed using a PAL rail system equipped with a 50/30 µm DVB/CAR/PDMS fiber (Supelco, Bellefonte, PA, USA). The incubation temperature was set to 60 °C with a 15 min preheating time, followed by a 30 min extraction and a 4 min desorption period. To prevent cross-contamination, fibers were conditioned at 250 °C for 10 min between samples. GC–MS analysis was conducted using an Agilent 7890 gas chromatograph coupled with a 5977B mass spectrometer equipped with a DB-Wax column. Samples were injected in split mode (50:1). Helium was used as the carrier gas with a front inlet purge flow of 3 mL min⁻¹ and a column flow rate of 1 mL min⁻¹. The initial oven temperature was held at 40 °C for 4 min, then increased to 245 °C at 5 °C min⁻¹ and held for 5 min. The injection port, transfer line, ion source, and quadrupole temperatures were set to 250 °C, 250 °C, 230 °C, and 150 °C, respectively. Electron impact ionization was performed at 70 eV. Mass spectra were acquired in scan mode over an m/z range of 20–400 with no solvent delay.

Raw data processing, including peak extraction, baseline correction, alignment, deconvolution, peak identification, integration, and spectral matching against the NIST database, was performed using Chroma TOF 4.3X software (LECO Corporation, St. Joseph, MI, USA).

### Statistical analysis

Student’s *t*-test was used to analyze aroma component data (Excel, Microsoft, USA). Orthogonal partial least squares–discriminant analysis (OPLS–DA) was conducted using SIMCA software (Version 14.1, Umetrics, Umeå, Sweden), and variable importance in projection (VIP) values were calculated from the first principal component to identify significant variables. Venn diagrams were generated using online tools (https://www.omicshare.com/tools/Home/Soft/venn). Figures were prepared with GraphPad Prism 8.4.3 and Origin 2018b. To avoid overly stringent filtering that would exclude biologically relevant candidates in this exploratory study, we used uncorrected *P* < 0.05 for initial screening. Nevertheless, this approach increases the risk of false positives.

## Results

### VOCs in jasmine buds and flowers

A total of 319 and 552 compounds were detected in commercial jasmine buds and opened flowers (Supplementary Materials 1), respectively. The variety and total relative abundance of volatile components increased significantly after flowering. Statistical analysis indicated that the major aroma components were aldehydes, alcohols, esters, acids, ketones, and hydrocarbons. In buds, aldehydes (11.02–41.92%), alcohols (12.37–39.06%), and hydrocarbons (2.09–7.38%) accounted for higher relative proportions. In opened flowers, alcohols (16.82–32.91%), hydrocarbons (15.26–21.87%), and esters (14.81–19.55%) were predominant, while the relative content of aldehydes decreased to 5.07–9.48%. The proportion of alcohols remained relatively stable across stages. Excluding unidentified substances, the top 3% most abundant compounds in both groups are listed in Table S2 & S3.

### OPLS-DA of aroma VOCs showed clear discrimination between the two grades of jasmine buds and flowers

**Fig. 1:**
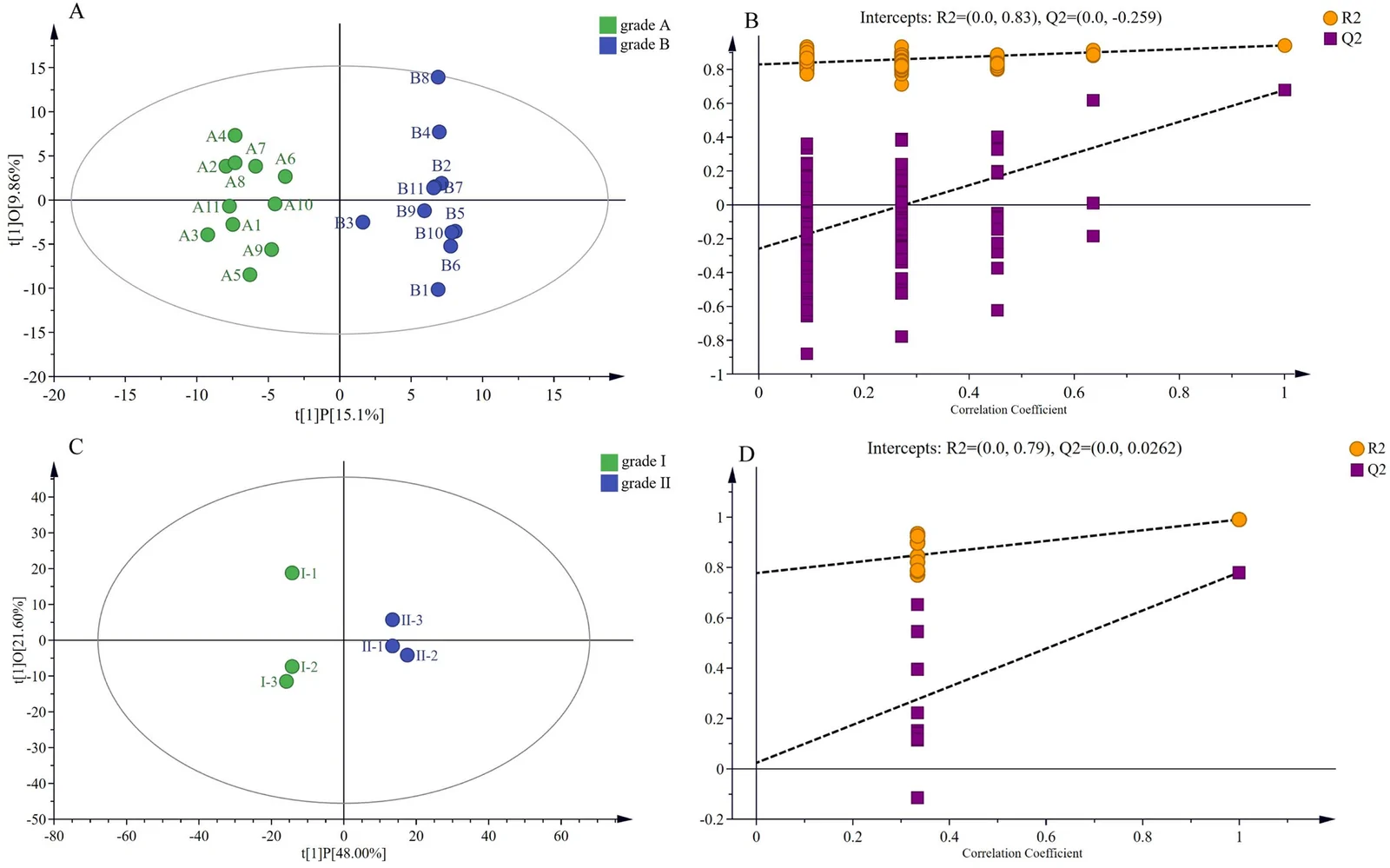
Orthogonal partial least squares–discriminant analysis (OPLS-DA) of VOCs in jasmine buds/flowers. (**A**) Score plot of bud grades (grade A vs. grade B). (**B**) Permutation test validation for the bud model. (**C**) Score plot of opened flower grades (grade I vs. grade II). (**D**) Permutation test validation for the opened flower model. All samples fell within the 95% confidence ellipse. Permutation tests (*n* = 200) confirmed model robustness and absence of overfitting. R^2^ represents the fraction of the variance in the Y-variable (group membership) explained by the model, and Q2 represents the predictive ability of the model.

Bud samples were graded according to grower standards into grade A and grade B. OPLS-DA performed on all aroma components revealed clear separation between the two grades (Fig. 1A). All samples fell within the 95% confidence interval. Permutation tests confirmed the model’s robustness, reliability, and absence of overfitting (Fig. 1B). Similarly, significant differences were observed between grade I and grade II opened flowers (Fig. 1C and D).

### Discriminant analysis of aroma compounds between the two grades

**Fig. 2:**
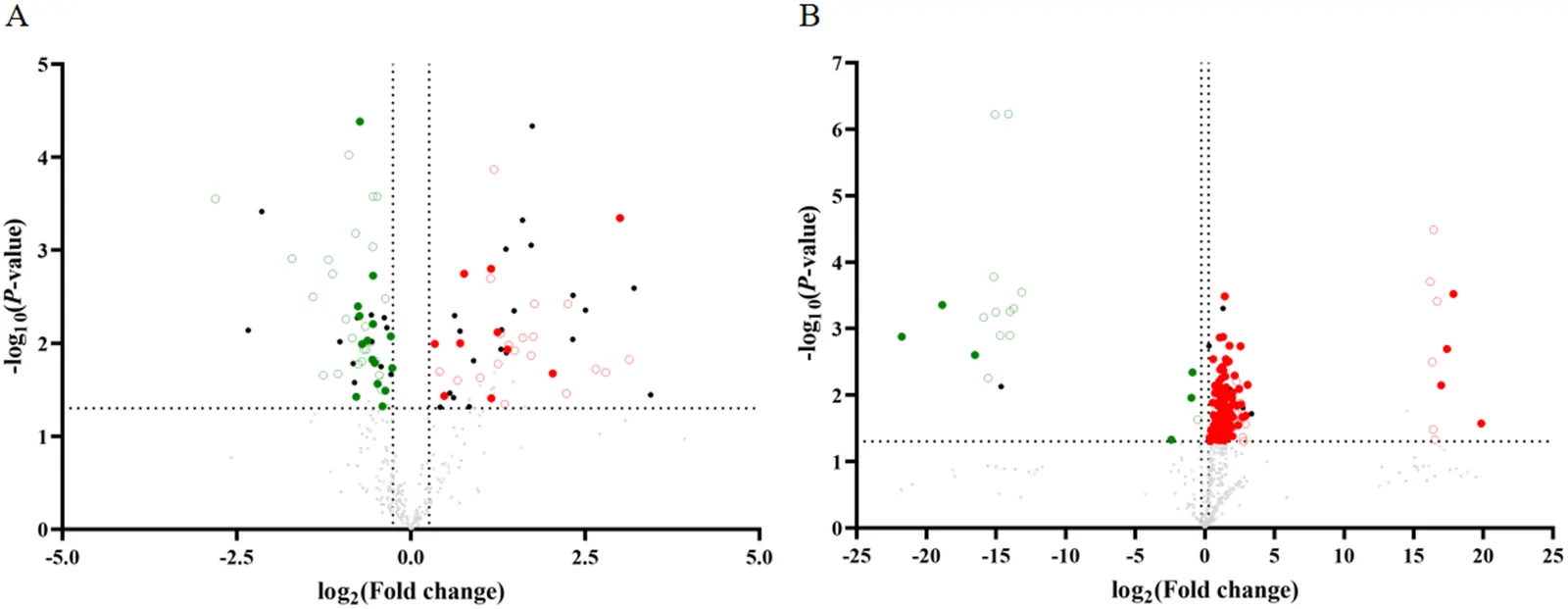
Volcano plots of differential VOCs between jasmine buds/flower grades. (**A**) Comparison of grade A vs. grade B buds. (**B**) Comparison of grade I vs. grade II opened flowers. Gray dots: not significant (*P* ≥ 0.05 or VIP ≤ 1); black points: significant but unidentified; open red/green circles: significant with relative content < 0.1 in all samples; solid red/green dots: significant with relative content ≥ 0.1 in at least one grade. Selection criteria: VIP > 1, (FC > 1.2 or < 1/1.2), and *P* < 0.05.

Variables with VIP > 1, fold change > 1.2 (red dots) or < 1/1.2 (green dots), and *P* < 0.05 were selected as discriminant compounds between grade A and grade B of jasmine buds, and between grade I and grade II of jasmine flowers (Fig. 2A and B). Unidentified compounds are marked in black, and differential metabolites with low relative abundance (< 0.1 in all grades) are shown as open circles. 10 compounds were significantly higher in grade A, and 15 were higher in grade B of jasmine buds (Fig. 2A). In opened flowers, 128 compounds were significantly higher in grade I, and 6 were higher in grade II of jasmine flowers (Fig. 2B).

**Fig. 3:**
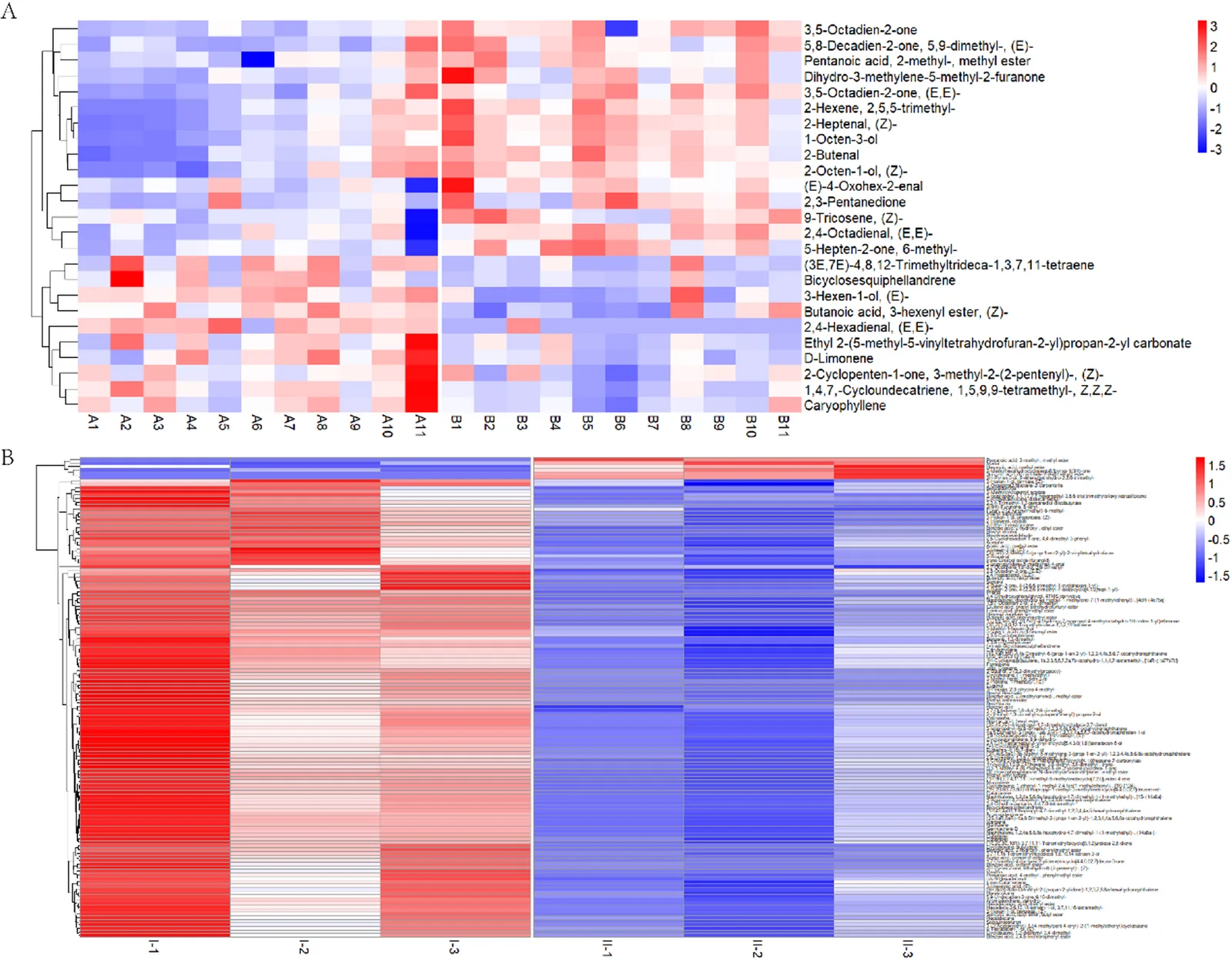
Hierarchical clustering heatmaps of differential volatile compounds. (**A**) grade A vs. grade B buds. (**B**) grade I vs. grade II opened flowers. Red indicates relative content above the mean; blue indicates below the mean. Each row represents a compound, and each column represents a sample.

Hierarchical clustering analysis was performed on the significant differential aroma components identified in the volcano plots, and heatmaps were generated for both bud and opened flower grades (Fig. 3, Supplementary Materials 2). Red indicates a relative content above the mean, and blue indicates below the mean. Among the 10 VOCs with significantly higher relative abundance in grade A (Fig. 3A), six showed a fold change greater than 2. However, except for *Z*,*Z*,*Z*−1,4,7-Cycloundecatriene-1,5,9,9-tetramethyl, which had an average relative abundance of 0.67, the relative abundances of the other VOCs were all below 0.3. Meanwhile, (*E*)−3-Hexen-1-ol showed the highest relative abundance among the differential metabolites (2.64 in grade A and 2.09 in grade B) with a 1.27-fold difference between the two grades. Among the 15 VOCs significantly decreased in grade A, the fold changes were all below 2; (*E*)−5,8-Decadien-2-one- 5,9-dimethyl and 3,5-Octadien-2-one had fold changes of 1.22 and 1.54, respectively, and average relative abundances > 1.

In opened flowers (Fig. 3B), more components showed higher relative abundances in grade I than in grade II, with overall higher abundance in both grades. Among the top 10 VOCs by relative abundance in grades I and II (Table S4), Benzyl alcohol and (*E*)−3-Hexen-1-ol were the two major alcohols, with levels in grade I more than double those in grade II. New components such as Farnesene were detected in opened flowers. While fewer VOCs were present in buds, opened flowers released a higher diversity and relative abundance of volatile compounds.

### Potential marker analysis for different jasmine flower grades

**Fig. 4:**
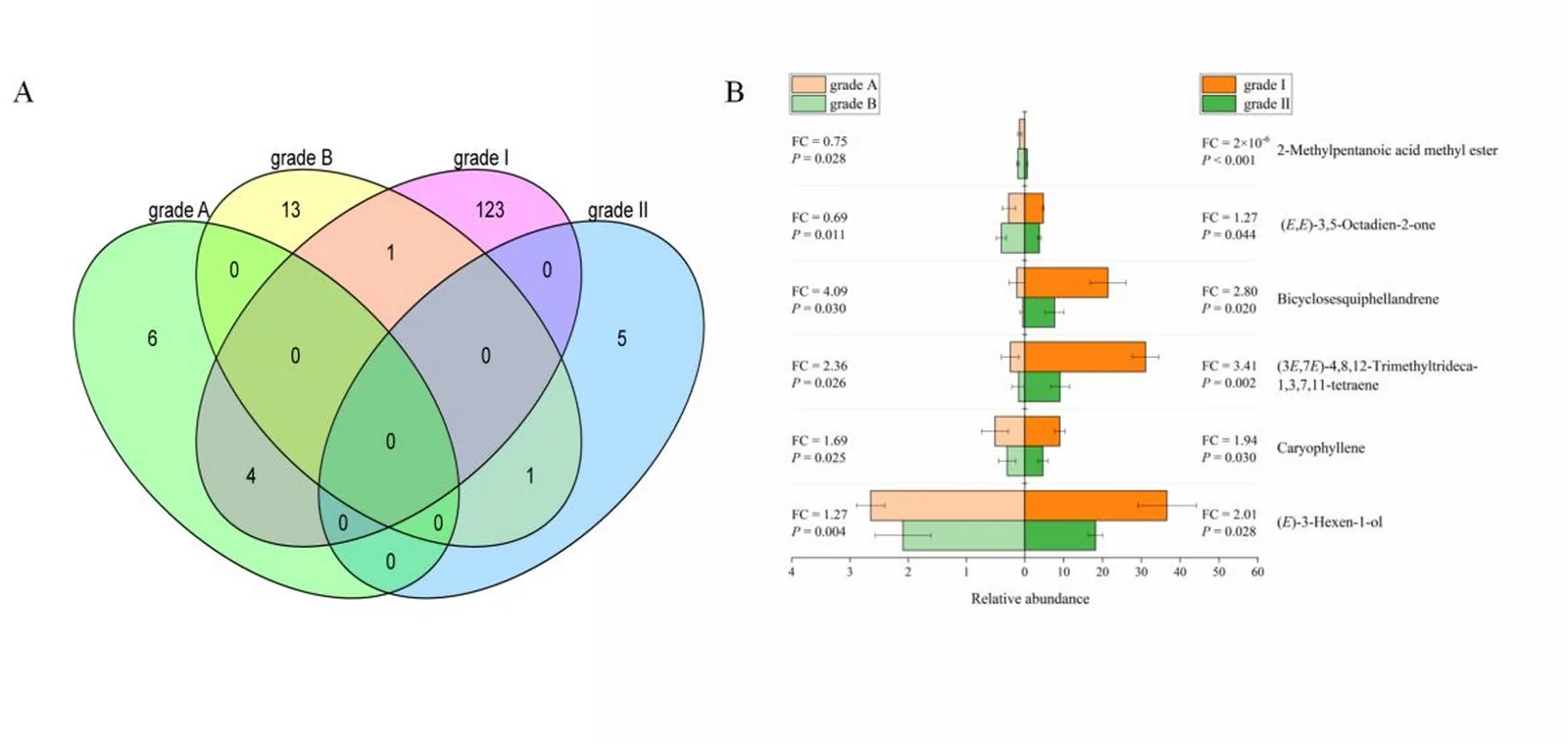
Common differential compounds across grade comparisons. (**A**) Venn diagram of differential VOC species across different grades. (**B**) Relative abundances and fold changes of compounds that are common to multiple grades. Error bars represent standard deviation (grade A&B: *n* = 11, grade I&II: *n* = 3).

A Venn diagram was constructed to identify common differential components across grades (Fig. 4A). Four compounds were significantly higher in both grade A buds and grade I flowers, while only one compound was higher in both grade B buds and grade II flowers. One compound was higher in both grade B buds and grade I flowers.

The relative abundances and fold changes of the intersecting compounds are shown in Fig. 4B. 2-Methylpentanoic acid methyl ester was higher in grade B buds and grade II flowers but exhibited low relative abundances (0.12 and 0.64, respectively). (*E*,*E*)−3,5-Octadien-2-one was higher in grade B buds and grade I flowers, with moderate relative content. The four compounds of Bicyclosesquiphellandrene, (3*E*,7*E*)−4,8,12-Trimethyltrideca-1,3,7,11-tetraene, Caryophyllene, and (*E*)−3-Hexen-1-ol indicated a consistent positive association with higher quality before and after jasmine flowering. All four showed high relative content and significant differences between grades within the same type. Among them, (*E*)−3-Hexen-1-ol displayed the highest relative abundance across all sample grades and showed significant inter-grade differences (Fig. 4B).

Based on the above differential analysis, six significantly different VOCs were finally selected. Among these, the relative abundances of (*E*)−3-Hexen-1-ol, Caryophyllene and (*E*,*E*)−3,5-Octadien-2-one were higher in both grade A and grade B. To further translate this predictive potential into a practical tool, we developed a simple algebraic formula based on these three robust markers that distinguishes the eventual post-anthesis grade solely from bud VOC data. In addition to (*E*)−3-Hexen-1-ol, Caryophyllene showed significantly higher levels in grade A and was consistently elevated in grade I. Conversely, (*E*,*E*)−3,5-Octadien-2-one was significantly lower in grade A but higher in grade I. Accordingly, we constructed the following ratio:

$$\:{\mathrm{S}} = \frac{{[{\mathrm{(E}}){\text{ - 3 - Hexen - 1 - ol}}] \times [{\mathrm{Caryophyllene}}]}}{{[({\mathrm{E,E}}){\text{ - 3}},{\text{5 - Octadien - 2 - one}}]^{{\mathrm{2}}} }}$$

S value: standard values for grade classification.

[(*E*)−3-Hexen-1-ol]: the relative abundance of (*E*)−3-Hexen-1-ol;

[Caryophyllene]: the relative abundance of Caryophyllene;

[(*E*,*E*)−3,5-Octadien-2-one]: the relative abundance of (*E*,*E*)−3,5-Octadien-2-one.

The denominator was squared to amplify the negative contribution of the inferior-grade marker while reducing the impact of measurement noise. Using the measured peak areas from the bud samples, S values for the grade A ranged from 9.93 to 15.21, whereas those for the grade B ranged from 4.15 to 9.23, with no overlap. Therefore, a simple decision rule applies: S > 9.5 predicts grade I, while S < 9.5 predicts grade II.

## Discussion

This study provides a comprehensive volatilomic analysis of commercially graded jasmine flowers, revealing distinct chemical signatures associated with different quality tiers. The findings provide a biochemical rationale for existing empirical grading practices, while identifying specific VOCs with potential as objective quality markers. However, the results are largely correlative, and further validation using independent datasets and absolute quantification is required.

The significant expansion in VOC diversity and total abundance post-anthesis, transitioning from an aldehyde-rich profile in buds to a dominant blend of alcohols, esters, and hydrocarbons in open flowers, aligns with the ecological function of floral scent in pollinator attraction^20^. This shift likely involves the coordinated activation of biosynthetic pathways, such as the lipoxygenase pathway leading to green leaf volatiles (e.g., (*E*)−3-Hexen-1-ol) and the shikimate/phenylpropanoid pathway yielding aromatic compounds like benzyl alcohol, upon flower opening^19,21^. The OPLS-DA models successfully discriminated grades based on these VOC profiles, confirming that chemical composition is a robust and quantifiable indicator of the phenotypic differences perceived by growers and industry.

For bud grading (A vs. B), the identification of (*E*)−3-Hexen-1-ol as a key differentiating compound is particularly noteworthy. Its higher concentration in grade A buds suggests a link between this “green” volatile and superior plant vigor or physiological status. (*E*)−3-Hexen-1-ol is often associated with plant defense responses and is a precursor for other aroma compounds. Its level may, therefore, reflect the overall health and metabolic activity of the plant, making it a promising predictive marker for the aromatic potential of buds^22,23^. Meanwhile, Caryophyllene and (*E*,*E*)−3,5-Octadien-2-one emerged as equally critical discriminators in bud grading, both showing high relative abundances.

Regarding open flowers, the substantial enrichment of key odorants—most prominently benzyl alcohol, (*E*)−3-Hexen-1-ol, and *α*-Farnesene—in mechanically sieved grade I flowers validates the empirical sieving method from a chemical perspective. These compounds are central to the characteristic sweet, floral, and fresh notes of jasmine^24^. Notably, *α*-Farnesene and Benzyl alcohol are well-established contributors to the prized fragrance of jasmine absolute and tea^25^. Their concurrent elevation in grade I flowers directly correlates with the sensory requirements for high-quality jasmine tea scenting. The fact that the sieving process (10 × 10 mm) effectively segregates flowers with this superior chemical profile underscores the practical utility of this physical method when calibrated correctly.

Based on the three selected markers—(*E*)−3-Hexen-1-ol, Caryophyllene, and (*E*,*E*)−3,5-Octadien-2-one—we developed a simple S-score formula. The formula requires only three readily detectable VOCs, offering a cost-effective, data-driven alternative to subjective visual or olfactory assessment of buds. However, the S-score model has several limitations. The formula and its cut-off value (S > 9.5) were developed and evaluated using the same dataset (*n* = 11 per bud grade). No independent validation or cross-validation was performed. Therefore, the model should be considered preliminary. While it achieves complete separation within this dataset, its performance on new samples from different seasons, growing regions, or production batches remains to be tested. We strongly recommend external validation before any practical application in jasmine grading.

## Conclusions

The study represents the first comprehensive determination of the aroma components of the jasmine flower buds and opened flowers, and clearly demonstrates the differences in two grades of jasmine flowers in two types. By comprehensively analyzing the differences in aroma components of commercial jasmine flowers in different periods, we identified three potential markers: (*E*)−3-Hexen-1-ol, Caryophyllene, and (*E*,*E*)−3,5-Octadien-2-one. Based on these markers, we developed a simple algebraic formula to predict the eventual quality of jasmine flowers solely from bud VOC data. Using a decision rule of S > 9.5 for high grade (A/I) and S < 9.5 for low grade (B/II), the formula achieved complete separation between grades without overlap. This S-score approach provides an objective, data-driven alternative to subjective visual or olfactory assessment. However, the model was developed using a single dataset without external validation; the threshold should therefore be considered preliminary and requires independent verification across different seasons, regions, or production batches before practical application.

## Supplementary Information

Below is the link to the electronic supplementary material.

Supplementary Material 1

Supplementary Material 2

Supplementary Material 3

## Data Availability

The datasets used and/or analysed during the current study available from the corresponding author on reasonable request.
