# Supplementary material for: Discriminant compounds and a predictive S-score for the manual grading standard of Jasminum sambac
Source: Sci Rep. 2026 Jun 22;16:28502. doi: 10.1038/s41598-026-57149-2 (PMC13572483; doi:10.1038/s41598-026-57149-2)
Supplement: Supplementary file 1 [file 41598_2026_57149_MOESM3_ESM.docx]

Table S1 VOCs with the top 3% relative content (%) in grade A and B.

| Peaks | RT | A1 | A2 | A3 | A4 | A5 | A6 | A7 | A8 | A9 | A10 | A11 | Mean±SD | B1 | B2 | B3 | B4 | B5 | B6 | B7 | B8 | B9 | B10 | B11 | Mean±SD |
| --- | --- | --- | --- | --- | --- | --- | --- | --- | --- | --- | --- | --- | --- | --- | --- | --- | --- | --- | --- | --- | --- | --- | --- | --- | --- |
| (*E*)-2-Hexenal | 11.5 | 23.99 | 27.54 | 25.28 | 16.38 | 34.80 | 22.29 | 22.54 | 15.33 | 26.37 | 19.37 | 13.78 | 22.51±5.85 | 0.00 | 23.99 | 30.20 | 25.50 | 30.84 | 35.56 | 31.49 | 14.13 | 32.75 | 30.97 | 29.41 | 25.90±9.84 |
| Linalool | 20.56 | 7.81 | 0.00 | 10.88 | 27.58 | 5.77 | 17.83 | 21.78 | 24.69 | 10.87 | 18.53 | 30.45 | 16.02±9.25 | 9.33 | 13.26 | 9.73 | 18.86 | 3.29 | 0.58 | 4.88 | 22.44 | 7.95 | 7.03 | 5.25 | 9.33±6.29 |
| Benzaldehyde | 19.67 | 6.66 | 6.22 | 5.73 | 5.12 | 5.84 | 5.71 | 4.27 | 4.84 | 7.00 | 5.31 | 7.83 | 5.87±0.97 | 6.91 | 5.86 | 7.28 | 5.13 | 5.39 | 5.44 | 5.21 | 3.52 | 5.01 | 5.52 | 8.17 | 5.77±1.21 |
| Hexanal | 7.37 | 2.80 | 4.54 | 3.05 | 2.31 | 4.74 | 2.90 | 2.97 | 2.01 | 3.21 | 2.39 | 1.72 | 2.97±0.90 | 0.00 | 3.30 | 4.21 | 3.49 | 5.03 | 4.56 | 5.08 | 0.00 | 3.34 | 2.73 | 3.29 | 3.18±1.67 |
| (*E*)-3-Hexen-1-ol | 16.31 | 3.59 | 3.48 | 2.61 | 2.96 | 2.74 | 3.23 | 3.27 | 2.31 | 2.55 | 2.84 | 2.23 | 2.89±0.44 | 4.39 | 2.03 | 1.83 | 1.68 | 2.08 | 2.57 | 2.48 | 4.40 | 1.72 | 2.50 | 2.47 | 2.56±0.92 |
| Benzeneacetaldehyde | 22.56 | 2.56 | 3.96 | 2.15 | 2.53 | 2.08 | 2.59 | 2.98 | 2.25 | 2.60 | 2.51 | 1.87 | 2.55±0.53 | 4.76 | 2.60 | 1.83 | 2.15 | 2.60 | 2.35 | 2.65 | 2.34 | 2.38 | 3.03 | 1.72 | 2.58±0.77 |
| octamethyl-Cyclotetrasiloxane | 5.43 | 2.41 | 2.31 | 1.97 | 1.77 | 2.03 | 1.91 | 1.85 | 2.13 | 2.36 | 2.26 | 1.83 | 2.08±0.22 | 0.00 | 2.73 | 2.02 | 2.13 | 2.95 | 3.23 | 2.74 | 2.48 | 2.47 | 2.89 | 2.69 | 2.39±0.83 |
| Methyl Alcohol | 3.25 | 2.20 | 1.97 | 1.94 | 1.61 | 2.24 | 1.54 | 1.68 | 1.59 | 1.96 | 1.87 | 1.33 | 1.81±0.27 | 4.00 | 2.26 | 1.69 | 1.61 | 2.59 | 2.46 | 2.17 | 1.43 | 1.88 | 1.76 | 1.54 | 2.13±0.70 |
| Hexanoic acid | 27.54 | 3.58 | 0.44 | 4.24 | 0.88 | 0.70 | 0.19 | 0.51 | 2.35 | 2.82 | 3.07 | 1.70 | 1.86±1.36 | 5.31 | 0.42 | 2.22 | 1.02 | 2.02 | 0.12 | 1.84 | 0.00 | 3.87 | 2.20 | 0.00 | 1.73±1.62 |
| 2-Octanone | 13.38 | 2.08 | 1.85 | 1.72 | 1.58 | 1.72 | 1.58 | 1.54 | 1.47 | 1.81 | 1.63 | 1.18 | 1.65±0.22 | 0.00 | 1.89 | 1.52 | 1.59 | 1.91 | 2.13 | 2.01 | 2.06 | 1.59 | 1.64 | 1.94 | 1.66±0.56 |

Table S2 VOCs with the top 3% relative content (%) in grade I and II.

| Peaks | RT | I1 | I2 | I3 | Mean±SD | II1 | II2 | II3 | Mean±SD |
| --- | --- | --- | --- | --- | --- | --- | --- | --- | --- |
| Linalool | 20.18 | 17.73 | 8.96 | 16.29 | 14.33±3.84 | 17.99 | 0.75 | 16.43 | 11.73±7.78 |
| Benzyl alcohol | 27.42 | 9.04 | 11.94 | 11.72 | 10.90±1.32 | 7.52 | 11.00 | 7.77 | 8.77±1.59 |
| Farnesene | 24.99 | 9.35 | 8.88 | 10.48 | 9.57±0.67 | 7.26 | 8.03 | 9.46 | 8.25±0.91 |
| Methyl salicylate | 25.11 | 3.41 | 5.21 | 4.84 | 4.49±0.78 | 3.52 | 3.47 | 2.76 | 3.25±0.35 |
| (*E*)-2-Hexenal | 10.97 | 2.30 | 3.43 | 2.69 | 2.81±0.47 | 3.62 | 4.91 | 3.48 | 4.00±0.64 |
| cis-3-Hexenyl cis-3-hexenoate | 24.18 | 1.58 | 2.34 | 4.48 | 2.80±1.23 | 2.80 | 3.02 | 3.21 | 3.01±0.17 |
| Benzaldehyde | 19.12 | 2.33 | 2.94 | 2.64 | 2.64±0.25 | 2.33 | 3.73 | 2.66 | 2.91±0.60 |
| Phenylmethyl ester acetic acid | 24.15 | 2.42 | 0.00 | 1.98 | 1.47±1.05 | 5.37 | 5.29 | 3.36 | 4.67±0.93 |
| (*Z*)-Butanoic acid-3-hexenyl ester | 17.82 | 3.00 | 3.19 | 0.00 | 2.06±1.46 | 3.04 | 3.62 | 2.97 | 3.21±0.29 |
| Hexanal | 6.93 | 0.64 | 1.08 | 0.75 | 0.82±0.19 | 1.24 | 1.67 | 0.87 | 1.26±0.33 |
| Myrcene | 9.10 | 0.74 | 1.05 | 0.73 | 0.84±0.15 | 1.09 | 1.40 | 0.98 | 1.16±0018 |
| Humulene | 22.85 | 0.95 | 0.90 | 1.19 | 1.01±0.13 | 0.62 | 0.73 | 0.87 | 0.74±0.10 |
| Methyl anthranilate | 34.16 | 1.37 | 0.93 | 1.31 | 1.20±0.19 | 0.34 | 0.28 | 0.33 | 0.31±0.03 |
| Methyl Alcohol | 3.00 | 0.57 | 0.78 | 0.75 | 0.70±0.09 | 1.01 | 1.13 | 0.79 | 0.98±0.14 |
| (*E*)-3-Hexen-1-ol | 15.85 | 0.62 | 1.09 | 0.85 | 0.85±0.19 | 0.65 | 0.85 | 0.66 | 0.72±0.09 |
| (*E*)-Butanoic acid-3-hexenyl ester | 23.79 | 0.73 | 0.68 | 0.89 | 0.77±0.09 | 0.40 | 0.41 | 0.62 | 0.48±0.10 |
| Germacrene D | 22.96 | 0.54 | 0.75 | 0.83 | 0.70±0.12 | 0.60 | 0.61 | 0.48 | 0.56±0.06 |

Table S3 Top 10 differential compounds by relative abundance in grades I and II.

| Peaks | I-1 | I-2 | I-3 | II-1 | II-2 | II-3 | mean I | mean II | Mean all | Fold Change |
| --- | --- | --- | --- | --- | --- | --- | --- | --- | --- | --- |
| Benzyl alcohol | 516.76 | 507.73 | 389.32 | 210.27 | 205.56 | 242.84 | 471.27 | 219.56 | 345.41 | 2.15 |
| Farnesene | 533.96 | 377.72 | 348.30 | 202.81 | 150.02 | 295.67 | 420.00 | 216.17 | 318.08 | 1.94 |
| Methyl salicylate | 195.12 | 221.66 | 160.97 | 98.46 | 64.88 | 86.35 | 192.58 | 83.23 | 137.91 | 2.31 |
| Benzaldehyde | 133.31 | 124.98 | 87.78 | 65.16 | 69.74 | 83.24 | 115.36 | 72.71 | 94.03 | 1.59 |
| Humulene | 54.11 | 38.31 | 39.69 | 17.35 | 13.69 | 27.20 | 44.04 | 19.41 | 31.72 | 2.27 |
| Methyl anthranilate | 78.42 | 39.62 | 43.42 | 9.49 | 5.14 | 10.21 | 53.82 | 8.28 | 31.05 | 6.50 |
| (*E*)-3-Hexen-1-ol | 35.22 | 46.45 | 28.19 | 18.24 | 15.94 | 20.48 | 36.62 | 18.22 | 27.42 | 2.01 |
| Germacrene D | 41.99 | 28.81 | 29.67 | 11.29 | 7.73 | 19.37 | 33.49 | 12.79 | 23.14 | 2.62 |
| 2-Ethyl-3-vinyloxirane | 30.65 | 31.68 | 27.61 | 16.73 | 11.46 | 14.92 | 29.98 | 14.37 | 22.18 | 2.09 |
| (3*E*,7*E*)-4,8,12-Trimethyltrideca-1,3,7,11-tetraene | 34.95 | 26.63 | 31.65 | 8.58 | 6.36 | 12.37 | 31.07 | 9.10 | 20.09 | 3.41 |
